# Obstructive Sleep Apnea in Obese Patients: a UK Population Analysis

**DOI:** 10.1007/s11695-020-05196-7

**Published:** 2021-01-09

**Authors:** S. Erridge, O. Moussa, C. McIntyre, A. Hariri, N. Tolley, B. Kotecha, S. Purkayastha

**Affiliations:** 1 Division of Surgery, Department of Surgery & Cancer, Imperial College London, Academic Surgical Unit, 10th Floor QEQM, St Mary’s Hospital, South Wharf Road, London, W2 1NY UK; 2 Department of Otolaryngology, Head and Neck Surgery, Imperial College Healthcare NHS Trust, London, W2 1NY UK; 3 Department of Otolaryngology, Head and Neck Surgery, Royal National Throat, Nose and Ear Hospital, Gray’s Inn Road, London, WC1X 8DA UK

**Keywords:** Obstructive sleep apnea, Obesity, Risk factors, Screening

## Abstract

**Background:**

Obstructive sleep apnea (OSA) is an increasingly common disorder associated with increased cardiovascular disease, mortality, reduced productivity, and an increased risk of road traffic accidents. A significant proportion of patients with OSA in the UK are undiagnosed. This study aims to identify risk factors for OSA in an obese cohort.

**Method:**

A population-based study was conducted of obese patients (BMI ≥ 30 kg/m^2^) from the Clinical Practice Research Datalink (CPRD). A logistic regression model was used to calculate odds ratios (ORs) for developing OSA according to other clinicopathological characteristics. Multivariate analysis was conducted of individual factors that affect the propensity to develop OSA. Statistical significance was defined as *p* < 0.050.

**Results:**

From 276,600 obese patients identified during a data extraction of the CPRD in July 2017, the prevalence of OSA was 5.4%. The following risk factors were found to be independently associated with increased likelihood of OSA: male sex (OR = 3.273; *p* < 0.001), BMI class II (OR = 1.640; *p* < 0.001), BMI class III (OR = 3.768; *p* < 0.001), smoking (OR = 1.179; *p* < 0.001), COPD (OR = 1.722; *p* < 0.001), GERD (OR = 1.557; *p* < 0.001), hypothyroidism (OR = 1.311; *p* < 0.001), acromegaly (OR = 3.543; *p* < 0.001), and benzodiazepine use (OR = 1.492; *p* < 0.001). Bariatric surgery was associated with reduced risk of OSA amongst this obese population (OR = 0.260; *p* < 0.001).

**Conclusions:**

In obese patients, there are numerous comorbidities that are associated with increased likelihood of OSA. These factors can help prompt clinicians to identify undiagnosed OSA. Bariatric surgery appears to be protective against developing OSA.

## Introduction

Obstructive sleep apnea (OSA) is the most common cause of sleep-disordered breathing and is estimated to affect a significant population of men (37%) and women (50%) [1, 2]*.* OSA is diagnosed through formal sleep studies and the severity of disease is classified according to the Apnea–Hypopnea Index (AHI) and symptom questionnaires [3, 4]. However, it is estimated that 80% of patients with OSA are undiagnosed and subsequently remain untreated [5, 6]. Obesity is associated with increased yearly incidence of OSA, with obese patients also affected by more severe disease [2, 7]. Obesity is a well-established risk factor for the development and progression of OSA, with increasing adiposity associated with a marked risk of developing OSA [7, 8]. The mechanism behind this is thought to be secondary to mass loading in the upper airway [2]. In addition to the well-recognized links with obesity, OSA has been associated with hypertension, cardiovascular disease (CVD), diabetes, chronic obstructive pulmonary disease (COPD), and depression [2].

OSA is characterized by recurrent episodes of upper airway collapse during sleep [9]. This can either be partial or complete, causing hypopnea or apnea respectively. Subsequently patients can experience oxygen desaturation, blood pressure and heart rate disturbance, as well as sleep fragmentation. Symptoms of OSA include daytime sleepiness, morning headaches, nocturia, irritability, somnolence, and fatigue [4]. Consequently, patients with OSA have poorer quality of life outcomes compared to healthy individuals [10]. Prompt recognition and appropriate therapy can ameliorate the neurobehavioral consequences and may also have favorable effects on cardiovascular and cerebrovascular health in moderate-severe disease [11]. Untreated OSA is independently associated with considerable long-term health risks [12]. OSA is associated with loss of productivity in the workplace and a higher incidence of road traffic accidents [13]. Recent data also suggests that moderate-to-severe OSA is independently associated with a significant increased risk of all-cause mortality [14].

Treatment of obesity and comorbid OSA with bariatric surgery has been demonstrated to improve OSA-specific outcomes, in addition to other metabolic outcomes and obesity-related mortality [15]. A recent meta-analysis of outcomes for sleep apnea following bariatric surgery demonstrated a significant improvement in weight loss and associated improvement in the severity of OSA post-operatively [16]. However, there is limited data to support a protective effect of bariatric surgery on developing OSA in patients without a pre-operative diagnosis.

The reasons behind poor detection of OSA amongst health professionals are likely multi-factorial, including lack of awareness, non-specific symptoms and signs, and healthcare avoidance by obese patients [17]. Furthering our understanding of the underlying mechanisms linking OSA to obese patients is essential in improving detection, treatment, and prevention of OSA. The aim of this study is to identify specific clinicopathological characteristics that affect the propensity for developing OSA in an obese cohort, including the effect of bariatric surgery, within a UK population.

## Methods

### Study Design

A population-based case-control study of obese patients (BMI ≥ 30 kg/m^2^) in a primary care setting was carried out utilizing the United Kingdom’s (UK) Clinical Practice Research Datalink (CPRD). The authors analyzed the effect of certain characteristics on the odds of developing OSA in obese patients.

The CPRD represents 8.5% of primary care practices across the UK and has been shown to be a representative of the general population [18]. End of follow-up was determined by one of the follow occurrences: change of practice, death, or final appointment recorded before data extraction.

### Ethics

Scientific approval was obtained from the Regulatory Agency’s Independent Scientific Advisory Committee, and ethical approval was attained through the Health Research Authority (IRAS Project ID: 203143, ISAC approval registration number 16_140R2).

### Patient and Data Selection

A consensus approach between two authors (S.E. and O.M.) was used to generate a list of clinically relevant “medcodes” to correlate demographics, diagnoses, and prescriptions. To assess the effect of BMI, the highest recorded weight was used to calculate maximum BMI [19]. BMI was categorized according to the World Health Organization’s obesity classification: class I (30.0 kg/m^2^ ≤ BMI < 35.0 kg/m^2^), class II (35.0 kg/m^2^ ≤ BMI < 40.0 kg/m^2^), and class III (BMI > 40.0 kg/m^2^) [19].

Coding data was examined to extract the following demographic variables: age and demographics, minimum weight, and maximum weight. In addition to this, comorbidities of obesity including hypertension, type II diabetes mellitus, and hyperlipidemia were sought. Previously associated risk factors for OSA that could be derived from the CPRD were identified including smoking, chronic obstructive pulmonary disease (COPD), gastro-esophageal reflux disease (GERD), chronic renal disease, hypothyroidism, acromegaly, craniofacial and upper airway anomalies, and benzodiazepine prescriptions. Medcodes were also extracted to identify patients who had been prescribed home continuous positive airway pressure (CPAP) therapy, which has been demonstrated to indicate the severity of OSA [18]. Scores from screening questionnaires such as STOP-Bang and Epworth Sleepiness Score were excluded as they do not form part of the diagnostic criteria for OSA, and their reliability for detecting confirmed disease is low [20].

Patients who had bariatric surgery, as recorded on the CPRD, were extracted within this dataset. Dates of surgery and OSA diagnosis were also extracted. From this, patients were coded as having had bariatric surgery only if the date of surgery was prior to a diagnosis of OSA, or if they were never diagnosed with OSA.

### Statistical Analysis

Demographic variables were analyzed using either an independent samples *t* test or chi-squared test dependent on whether the variables with continuous and normally distributed or categorical. Independent variables were examined for their association with OSA using a logistic regression model to calculate odds ratios (ORs) and corresponding 95% confidence intervals (95%CI). ORs with a *p* value < 0.050 on univariate analysis were used to define which factors should be incorporated into a multivariate analysis. Statistical significance was again defined using *p* value < 0.05. The same analysis was conducted on patients with OSA to determine which factors were associated with increased likelihood of continuous positive airway pressure (CPAP) requirement. All statistics were handled and analyzed using Statistical Package for Social Sciences (SPSS) (IBM Statistics version 24 SPSS Inc., (New York, IL), USA).

## Results

Initial data extraction identified 363,037 patients with a BMI ≥ 30 kg/m^2^. Exclusion criteria included patients with erroneous coding of a last clinical encounter later than date of extraction (22.07.2016), minimum weight less than 60 kg, maximum weight more than 300 kg, and patients with follow-up less than 5 years.

Data restrictions:

- Initial extract: 363,037
- Erroneous coding restriction: 363,021
- Weight restriction: 362,923
- Follow-up restriction: 276,210

There was a significant difference in mean age between obese patients with (61.3 ± 13.8) or without (61.0 ± 16.7; *p* = 0.035) a diagnosis of OSA. 59.8% (*n* = 8477) of the patients diagnosed with OSA were male, compared to 35.2% (*n* = 92,271; *p* < 0.001) of patients without OSA. A greater proportion of patients with OSA belonged to BMI class III (56.9%; *n* = 8059) than in the no OSA group (34.4%; *n* = 89,857; *p* < 0.001). Median follow-up was 147.0 months (range: 60.0–1034.0 months). Table 1 demonstrates the patients’ demographics and clinical characteristics in full.

**Table 1:** Demographics and clinical characteristics of study patients

	OSA	No OSA	*p* value
Subjects (*n*)	14,172	262,038	-
Age	61.3 ± 13.8	61.0 ± 16.7	0.035
Sex			< 0.001
Male	8477 (59.8%)	92,271 (35.2%)	
Female	5695 (40.2%)	169,767 (64.8%)	
Min BMI	34.3 ± 4.7	33.0 ± 3.8	< 0.001
Max BMI	42.7 ± 8.0	38.7 ± 6.4	< 0.001
BMI category			< 0.001
Class I	2395 (16.9%)	86,379 (33.0%)	
Class II	3718 (26.2%)	85,802 (32.7%)	
Class III	8059 (56.9%)	89,857 (34.3%)	
Bariatric surgery	55 (0.4%)	3031 (1.2%)	< 0.001
Hypertension	7742 (54.6%)	117,825 (45.0%)	< 0.001
Diabetes mellitus	5589 (39.4%)	65,311 (24.9%)	< 0.001
Hyperlipidemia	1976 (13.9%)	26,880 (10.3%)	< 0.001
Tobacco use	6029 (42.5%)	91,509 (34.9%)	< 0.001
COPD	1558 (11.0%)	13,686 (5.2%)	< 0.001
GERD	3199 (22.6%)	41,103 (15.7%)	< 0.001
Chronic renal disease	364 (2.6%)	4699 (1.8%)	< 0.001
Hypothyroidism	1602 (11.3%)	27,186 (10.4%)	< 0.001
Acromegaly	20 (0.1%)	81 (< 0.1%)	< 0.001
Craniofacial/upper airway anomalies	2 (< 0.1%)	37 (< 0.1%)	0.999
Benzodiazepine use	5620 (39.7%)	82,173 (31.4%)	< 0.001

*BMI* body mass index (kg/m^2^)

*COPD* chronic obstructive pulmonary disease

*GERD* gastro-esophageal reflux disease

### Prognostic Factors for Developing OSA

Table 2 demonstrates the results of the univariate analysis of variables analyzed for their effect on developing OSA. The following were all found to be associated with development of OSA; age greater than or equal to 60 years (OR = 1.094; 95%CI 1.057–1.132; *p* < 0.001), male sex (OR = 2.739; 95%CI 2.646–2.835; *p* < 0.001), BMI class II (OR = 1.563; 95%CI 1.483–1.647; *p* < 0.001), BMI class III (OR = 3.235; 95%CI 3.088–3.389; *p* < 0.001), hypertension (OR = 1.474; 95%CI 1.425–1.525; *p* < 0.001), diabetes mellitus (OR = 1.961; 95%CI 1.894–2.031; *p* < 0.001), hyperlipidemia (OR = 1.417; 95%CI 1.349–1.489; *p* < 0.001), smoking (OR = 1.380; 95%CI 1.333–1.428; *p* < 0.001), COPD (OR = 2.241; 95%CI 2.121–2.369; *p* < 0.001), GERD (OR = 1.567; 95%CI 1.504–1.632; *p* < 0.001), chronic renal disease (OR = 1.444; 95%CI 1.296–1.608; *p* < 0.001), hypothyroidism (OR = 1.101; 95%CI 1.044–1.161; *p* < 0.001), acromegaly (OR = 4.570; 95%CI 2.801–7.548; p < 0.001), and benzodiazepine use (OR = 1.438; 95%CI 1.389–1.489; *p* < 0.001). Bariatric surgery was associated with protection from developing OSA (OR = 0.333; 95%CI 0.255–0.435; *p* < 0.001).

**Table 2:** Univariate analysis of factors associated with developing OSA

Variable	*n*	OR (95%CI)	*p* value
Age			
< 60 years old	130,008	1	
≥ 60 years old	146,202	1.094 (1.057–1.132)	< 0.001
Sex			
Female	100,748	1	
Male	175,462	2.739 (2.646–2.835)	< 0.001
BMI category			
Class I	88,774	1	
Class II	89,520	1.563 (1.483–1.647)	< 0.001
Class III	97,916	3.235 (3.088–3.389)	< 0.001
Hypertension			
Absent	150,643	1	
Present	125,567	1.474 (1.425–1.525)	< 0.001
Diabetes mellitus			
Absent	205,310	1	
Present	70,900	1.961 (1.894–2.031)	< 0.001
Hyperlipidemia			
Absent	247,354	1	
Present	28,856	1.417 (1.349–1.489)	< 0.001
Tobacco			
Non-smoker	178,672	1	
Smoker	97,538	1.380 (1.333–1.428)	< 0.001
COPD			
No diagnosis	260,966	1	
Positive diagnosis	15,244	2.241 (2.121–2.369)	< 0.001
GERD			
No diagnosis	231,908	1	
Positive diagnosis	44,302	1.567 (1.504–1.632)	< 0.001
Chronic renal disease			
No diagnosis	271,147	1	
Positive diagnosis	5063	1.444 (1.296–1.608)	< 0.001
Hypothyroidism			
No diagnosis	247,422	1	
Positive diagnosis	28,788	1.101 (1.044–1.161)	< 0.001
Acromegaly			
No diagnosis	276,109	1	
Positive diagnosis	101	4.570 (2.801–7.548)	< 0.001
Craniofacial/upper airway anomalies			
No diagnosis	276,171	1	
Positive diagnosis	39	0.999 (0.241–4.147)	0.999
Benzodiazepines			
Not prescribed	188,417	1	
Prescribed	87,793	1.438 (1.389–1.489)	< 0.001
Bariatric surgery			
No surgery	273,124	1	
Surgery	3086	0.333 (0.255–0.435)	< 0.001

Variables significantly associated with OSA were included in a multivariate analysis as shown in Table 3. The following risk factors were found to be independently associated with increased likelihood of OSA: male sex (OR = 3.273; 95%CI 3.154–3.396; *p* < 0.001), BMI class II (OR = 1.640; 95%CI 1.556–1.739; *p* < 0.001), BMI class III (OR = 3.768; 95%CI 3.539–3.955; *p* < 0.001), hypertension (OR = 1.174; 95%CI 1.130–1.220; *p* < 0.001), diabetes mellitus (OR = 1.343; 95%CI 1.292–1.395; *p* < 0.001), hyperlipidemia (OR = 1.157; 95%CI 1.099–1.219; *p* < 0.001), smoking (OR = 1.179; 95%CI 1.138–1.223; *p* < 0.001), COPD (OR = 1.722; 95%CI 1.622–1.828; *p* < 0.001), GERD (OR = 1.557; 95%CI 1.493–1.625; *p* < 0.001), hypothyroidism (OR = 1.311 95%CI 1.239–1.387; *p* < 0.001), acromegaly (OR = 3.543; 95%CI 2.108–5.956; *p* < 0.001), and benzodiazepine use (OR = 1.492; 95%CI 1.439–1.548; *p* < 0.001). Bariatric surgery was independently associated with reduced prevalence of OSA amongst this obese population (OR = 0.260; 95%CI 0.199–0.340; *p* < 0.001).

**Table 3:** multivariate analysis of the risk factors associated with OSA

Variable	*n*	OR (95%CI)	*p* value
Age			
< 60 years old	130,008	1	
≥ 60 years old	146,202	0.932 (0.896–0.969)	< 0.001
Sex			
Female	100,748	1	
Male	175,462	3.273 (3.154–3.396)	< 0.001
BMI category			
Class I	88,774	1	
Class II	89,520	1.640 (1.556–1.739)	< 0.001
Class III	97,916	3.768 (3.539–3.955)	< 0.001
Hypertension			
Absent	150,643	1	
Present	125,567	1.174 (1.130–1.220)	< 0.001
Diabetes mellitus			
Absent	205,310	1	
Present	70,900	1.343 (1.292–1.395)	< 0.001
Hyperlipidemia			
Absent	247,354	1	
Present	28,856	1.157 (1.099–1.219)	< 0.001
Tobacco			
Non-smoker	178,672	1	
Smoker	97,538	1.179 (1.138–1.223)	< 0.001
COPD			
No diagnosis	260,966	1	
Positive diagnosis	15,244	1.722 (1.622–1.828)	< 0.001
GERD			
No diagnosis	231,908	1	
Positive diagnosis	44,302	1.557 (1.493–1.625)	< 0.001
Chronic renal disease			
No diagnosis	271,147	1	
Positive diagnosis	5063	1.088 (0.972–1.217)	0.143
Hypothyroidism			
No diagnosis	247,422	1	
Positive diagnosis	28,788	1.311 (1.239–1.387)	< 0.001
Acromegaly			
No diagnosis	276,109	1	
Positive diagnosis	101	3.543 (2.108–5.956)	< 0.001
Benzodiazepines			
Not prescribed	188,417	1	
Prescribed	87,793	1.492 (1.439–1.548)	< 0.001
Bariatric surgery			
No surgery	273,124	1	
Surgery	3086	0.260 (0.199–0.340)	< 0.001

### Risk Factors for CPAP Requirement in OSA

Univariate analysis of risk factors for CPAP requirement in OSA, a surrogate marker of severity, is shown in Table 4. OSA requiring CPAP was significantly associated with the following risk factors risk factors in this obese population: male sex (OR = 1.874; 95%CI 1.667–2.105; *p* < 0.001), BMI class II (OR = 1.588; 95%CI 1.311–1.922; *p* < 0.001), BMI class III (OR = 1.973; 95%CI 1.660–2.344; *p* < 0.001), hypertension (OR = 1.268; 95%CI 1.139–1.412; *p* < 0.001), diabetes mellitus (OR = 1.213; 95%CI 1.091–1.349; *p* < 0.001). Benzodiazepine use was associated with reduced CPAP requirement in OSA patients (OR = 0.753; 95%CI 0.673–0.841; *p* < 0.001).

**Table 4:** Univariate analysis of the requirement for CPAP in OSA

Variable	*n*	OR (95%CI)	*p* value
Age			
< 60 years old	6371	1	
≥ 60 years old	7801	1.036 (0.932–1.152)	0.515
Sex			
Female	5695	1	
Male	8477	1.874 (1.667–2.105)	< 0.001
BMI category			
Class I	2395	1	
Class II	3718	1.588 (1.311–1.922)	< 0.001
Class III	8059	1.973 (1.660–2.344)	< 0.001
Hypertension			
Absent	6430	1	
Present	7742	1.268 (1.139–1.412)	< 0.001
Diabetes mellitus			
Absent	8583	1	
Present	5589	1.213 (1.091–1.349)	< 0.001
Hyperlipidemia			
Absent	12,196	1	
Present	1976	1.081 (0.931–1.254)	0.306
Tobacco			
Non-smoker	8143	1	
Smoker	6029	1.087 (0.978–1.209)	0.122
COPD			
No diagnosis	12,614	1	
Positive diagnosis	1558	0.920 (0.774–1.094)	0.347
GERD			
No diagnosis	10,973	1	
Positive diagnosis	3199	1.017 (0.897–1.153)	0.797
Chronic renal disease			
No diagnosis	13,808	1	
Positive diagnosis	364	0.888 (0.627–1.258)	0.504
Hypothyroidism			
No diagnosis	12,570	1	
Positive diagnosis	1602	0.875 (0.736–1.041)	0.132
Acromegaly			
No diagnosis	14,152	1	
Positive diagnosis	20	1.433 (0.419–4.894)	0.566
Craniofacial/upper airway anomalies			
No diagnosis	14,170	1	
Positive diagnosis	2	N/A	0.999
Benzodiazepines			
Not prescribed	8552	1	
Prescribed	5620	0.753 (0.673–0.841)	< 0.001
Bariatric surgery			
No surgery	14,117	1	
Surgery	55	0.845 (0.381–1.870)	0.845

Multivariate analysis, as detailed in Table 5, found male sex (OR = 1.833; 95%CI 1.637–2.077; *p* < 0.001), BMI class II (OR = 1.543; 95%CI 1.273–1.870; *p* < 0.001), BMI class III (OR = 1.997; 95%CI 1.677–2.378; *p* < 0.001), hypertension (OR = 1.191; 95%CI 1.066–1.330; *p* = 0.002). Benzodiazepine use was again associated with reduced CPAP requirement in OSA patients (OR = 0.822; 95%CI 0.734–0.920; *p* = 0.001).

**Table 5:** Multivariate analysis CPAP requirement in OSA

Variable	*n*	OR (95%CI)	*p* value
Sex			
Female	5695	1	
Male	8477	1.844 (1.637–2.077)	< 0.001
BMI category			
Class I	2395	1	
Class II	3718	1.543 (1.273–1.870)	< 0.001
Class III	8059	1.997 (1.677–2.378)	< 0.001
Hypertension			
Absent	6430	1	
Present	7742	1.191 (1.066–1.330)	0.002
Diabetes mellitus			
Absent	8583	1	
Present	5589	1.042 (0.933–1.163)	0.471
Benzodiazepines			
Not prescribed	8552	1	
Prescribed	5620	0.822 (0.734–0.920)	0.001

## Discussion

This large-scale population analysis of obese patients from a primary care dataset demonstrates the relationships between different clinicopathological factors and the development of OSA. A number of risk factors that have previously shown an association with OSA such male sex, increasing BMI, smoking, COPD, GERD, hypothyroidism, acromegaly, and benzodiazepine prescriptions were again shown to confer a greater risk to patients. Presence of the metabolic sequelae of obesity was also demonstrated to be associated with increased propensity to acquire OSA. Bariatric surgery was shown to be associated with reduced probability of developing an OSA diagnosis.

These results suggest that increasing BMI beyond category I is independently associated with further risk of OSA. This is in keeping with findings from the seminal Wisconsin Sleep Cohort Study that demonstrated that one standard deviation increment of BMI measurement results in 4-fold increase in odds of sleep-disordered breathing [1]. This is in addition to a number of other population-based studies from different regions [21–24]. The mechanism is thought to be secondary to increased mass loading around airway structures from fat deposition, causing a reduction in airway patency. Neck circumference is subsequently the measure of adiposity that is most closely associated with OSA [1]. However, central adiposity is also thought to play a crucial role through reducing the functional residual capacity of the lungs, again affecting upper airway patency [8, 25]. Longitudinal studies of patients with sleep-disordered breathing have demonstrated that increases in body weight hasten the development of OSA and increase the severity of disease [26–28]. This is supported by the results demonstrating that those patients with a BMI in category II/III were more likely to require CPAP, a surrogate measure of severity.

This study also highlighted that bariatric surgery was associated with reduced likelihood of developing OSA. Bariatric surgery provides both weight-dependent and weight-independent effects to achieve health benefits to patients. As highlighted, the pathophysiology of OSA and sleep-disordered breathing is fundamentally secondary to increased adiposity in the majority of patients. A meta-analysis by Sarkhosh and colleagues found that close to 35% of patients undergoing bariatric surgery had OSA. Seventy-nine percent of these patients experienced resolution or improvement in sleep apnea [29]. A meta-analysis by Buchwald et al. that included a broader definition of OSA, including sleep-disordered breathing, found similarly high rates of resolution of resolution or improvement (83.6%) [30]. This study demonstrated that bariatric surgery is associated with fewer OSA diagnoses in an initially obese population. Whilst no previous study has investigated this effect, this finding is in keeping with the high prevalence of OSA resolution post-bariatric surgery [29]. Reduction in OSA in this post-operative group will also be associated with improved long-term health and economic outcomes.

A white paper from the American Academy of Sleep Medicine estimated that the cost of treating diagnosed OSA in 2015 was approximately $12.4 billion USD [31]. The cost of untreated OSA was also estimated at $149.6 billion USD incorporating associated productivity costs, healthcare expenses, motor vehicle accidents, and workplace accidents [31]. Coupled with the present study outcomes, bariatric surgery as an effective obesity treatment may confer improved long-term health and economic outcomes through reducing OSA incidence across global health systems.

This study was able to demonstrate risk factors that had an independent association with an OSA diagnosis. Men were found to be both at higher risk of OSA, and subsequently OSA with worse severity as indicated by home CPAP use. The reason for this is likely multifactorial as women are more likely to present with non-classical symptoms of fatigue and tiredness, rather than heavy snoring with witnessed apneic episodes [32]. Male bed partners of female patients having higher thresholds for OSA symptom perception compound this, making diagnosing female patients with OSA more difficult [33]. There is also a likely biological basis for this difference in disease prevalence including anatomical differences in the upper airways between both sexes and hormonal influences [34–36].

Abnormalities in craniofacial anatomy have previously been demonstrated to contribute to the pathogenesis of OSA and sleep-disordered breathing. Deviations from normal anatomy of the pharynx, glottis, and bones of the head and neck can increase the susceptibility for airway collapse and subsequent apneic episodes during sleep [36]. The results from this study were unable to show any relationship between diagnosed craniofacial abnormalities. This is however accounted for by the limitations of database studies, which are reliant on correct coding. The prevalence of craniofacial abnormalities detected in the population of this study is unlikely to be a true prevalence. Contrastingly acromegaly showed a significant association with OSA. Acromegaly, characterized by excessive endogenous growth hormone, can impair maintenance of airway patency during sleep by causing changes in the size and shape of the mandible and maxilla, as well as soft tissue thickening of the soft palate, uvula, and tongue [37].

The CPRD is the largest primary care database in the UK allowing large-scale population research as demonstrated in this study [18]. The CPRD has previously been shown to be representative of the UK population, ensuring external validity of this and other analyses from the CPRD [38]. A limitation of database research however is that it is reliant on accurate coding of clinical and demographic data. To minimize this effect, exclusion criteria were used to limit the sample by weight, data entry dates, and patients with follow-up less than 5 years. OSA, as mentioned, is formally diagnosed by sleep studies in a specialist secondary or tertiary care setting. As the CPRD is a primary care database, coding of OSA diagnosis is likely to be less reliable than conditions where the diagnosis is made in primary care, leading to underestimation of the prevalence of OSA amongst the study population. Additionally, CPAP pressures were not recorded in the CPRD, which would help further differentiate disease severity between patients. The underestimation of OSA, as well as other variables, would however bias results towards the null. In practice, this means that the findings from this study would under-represent the strength of any associations found rather than overstate any relationships. Similarly deriving interpretations of severity of disease is difficult from the CPRD as AHI scores are not reliably represented by any “medcode” within the datalink. In this study, prescriptions for home CPAP were used as a surrogate marker for disease severity. This is a useful marker of severity as the evidence to support CPAP in asymptomatic OSA is weak, and CPAP titration is subsequently conducted in patients with excessive daytime sleepiness or fatigue. UK guidance for OSA is currently in development and subsequently the accuracy of using CPAP prescriptions to indicate disease severity is limited. Additionally, CPAP pressures were not recorded in the CPRD, which would help further differentiate between disease severities in patients.

## Conclusion

This population study of primary care patients with obesity in the UK has highlighted that increasing BMI further increases the likelihood for patients to develop OSA. Importantly, bariatric surgery is independently associated with reduced possibility of developing OSA. This may be an important factor in the long-term health benefits of obesity surgery [39]. OSA diagnosis rates are poor. By identifying the clinicopathological characteristics that increase the propensity for patients to develop OSA, these results aid primary care providers and non-specialists in obesity and/or respiratory medicine to identify which obese patients are more likely to develop OSA. If utilized, this could subsequently be used to prompt screening, initiate investigation, and improve access to treatment and bariatric surgery for obese patients under their care.
